# Expression and localization of aromatase during fetal mouse testis development

**DOI:** 10.1186/2051-4190-23-12

**Published:** 2013-12-01

**Authors:** Caroline Borday, Jorge Merlet, Chrystèle Racine, René Habert

**Affiliations:** Laboratory of Development of the Gonads, Unit of Stem Cells and Radiation, Univ. Paris Diderot, Sorbonne Paris Cité, BP 6, 92265 Fontenay-aux-Roses, France; CEA, DSV, iRCM, SCSR, LDG, 92265 Fontenay-aux-Roses, France; Unit of Stem Cells and Radiation, LDG / SCSR / iRCM / DSV, INSERM, Centre CEA, BP6, Unité 967, F-92265 Fontenay aux Roses, France

**Keywords:** Cyp19a1, Aromatase, Testis, Fetus, Mouse, Gonocytes, Development, Endocrine disruptors, Leydig cells, Souris, Développement, Perturbateurs endocriniens, Cellules de Leydig, Cyp19a1, Aromatase, Testis, Foetus, Souris, Gonocytes, Développement, Perturbateurs endocriniens, Cellules de Leydig

## Abstract

**Background:**

Both androgens and estrogens are necessary to ensure proper testis development and function. Studies on endocrine disruptors have highlighted the importance of maintaining the balance between androgens and estrogens during fetal development, when testis is highly sensitive to environmental disturbances. This balance is regulated mainly through an enzymatic cascade that converts irreversibly androgens into estrogens. The most important and regulated component of this cascade is its terminal enzyme: the cytochrome p450 19A1 (aromatase hereafter). This study was conducted to improve our knowledge about its expression during mouse testis development.

**Findings:**

By RT-PCR and western blotting, we show that full-length aromatase is expressed as early as 12.5 day post-coitum (dpc) with maximal expression at 17.5 dpc. Two additional truncated transcripts were also detected by RT-PCR. Immunostaining of fetal testis sections and of gonocyte-enriched cell cultures revealed that aromatase is strongly expressed in fetal Leydig cells and at variable levels in gonocytes. Conversely, it was not detected in Sertoli cells.

**Conclusions:**

This study shows for the first time that i) aromatase is expressed from the early stages of fetal testis development, ii) it is expressed in mouse gonocytes suggesting that fetal germ cells exert an endocrine function in this species and that the ratio between estrogens and androgens may be higher inside gonocytes than in the interstitial fluid. Furthermore, we emphasized a species-specific cell localization. Indeed, previous works found that in the rat aromatase is expressed both in Sertoli and Leydig cells. We propose to take into account this species difference as a new concept to better understand the changes in susceptibility to Endocrine Disruptors from one species to another.

**Electronic supplementary material:**

The online version of this article (doi:10.1186/2051-4190-23-12) contains supplementary material, which is available to authorized users.

## Résumé

Les androgènes et les oestrogènes sont indispensables au développement et aux fonctions du testicule. Le testicule est particulièrement sensible aux perturbateurs endocriniens pendant le développement fœtal et beaucoup de perturbateurs endocriniens agissent en modifiant la balance oestrogènes/androgènes. Physiologiquement, cette balance est régulée par une cascade enzymatique qui convertit irréversiblement les androgènes en oestrogènes. Le composant principal de cette cascade est le cytochrome p450 19A1 (appelé couramment aromatase). Le but de ce travail a été d’étudier l’expression de l’aromatase testiculaire au cours du développement fœtal chez la souris.

En utilisant une approche par RT-PCR et par western blot, nous avons montré que l’aromatase est exprimée dès 12,5 jours post-conception (jpc) et que l’expression est maximum à 17,5 jpc. Deux transcripts tronqués ont également été détectés par RT-PCR. La localisation cellulaire de l’aromatase a été étudiée par immunohistologie et par immunomarquage après séparation des cellules testiculaires. Cette enzyme est très fortement exprimée dans les cellules de Leydig fœtales. Elle est également exprimée dans les gonocytes mais plus faiblement et à un niveau variable selon les cellules. En revanche, elle est indétectable dans les cellules de Sertoli.

En conclusion, cette étude montre pour la première fois chez la souris que 1) l’aromatase est exprimée dès le début de l’ontogenèse testiculaire, 2) elle est exprimée dans les gonocytes suggérant que ces cellules interviennent dans l’endocrinologie testiculaire et que le rapport oestrogènes/androgènes est plus important dans les gonocytes que dans le liquide interstitiel. En outre, on sait que, chez le fœtus de rat l’aromatase est essentiellement exprimée par les cellules de Sertoli. Nous proposons de prendre en compte cette différence inter-espèces comme un nouveau concept pour comprendre les différences de sensibilité aux perturbateurs endocriniens d’une espèce à l’autre.

## Findings

### Ontogenesis of cytochrome P450 aromatase expression in the mouse testis during fetal development

C57BL/6 mice bred in our animal facility were housed under controlled photoperiod conditions (lights from 08:00 to 20:00 h) with commercial food and tap water supplied ad libitum, as previously described [1, 2]. The day after overnight mating was counted as 0.5 day post-coitum (dpc). The animal facility is licensed by the French Ministry of Agriculture (agreement N°B92-032-02). All animal experiments were supervised by Pr. René Habert (agreement delivered by the French Ministry of Agriculture for animal experimentation N°92-191) in compliance with the NIH Guide for Care and Use of Laboratory Animals.

**Figure 1:**
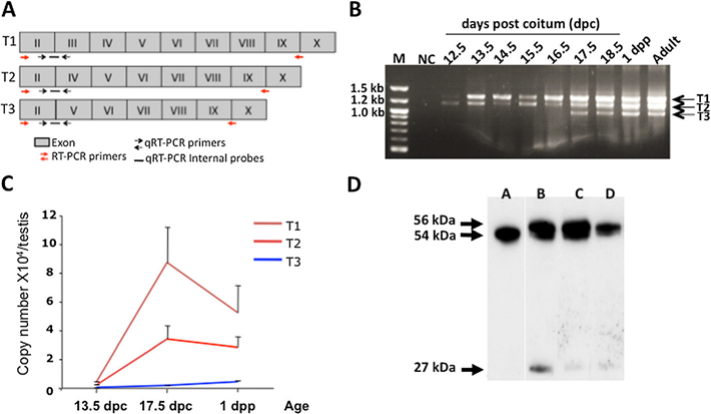
**Aromatase expression in mouse fetal testis. A.** Scheme showing the different testis aromatase variants (T1, T2 and T3). Aromatase exons from exon 2 to exon 10 are represented (gray boxes) for each transcript variant T1, T2 and T3. For each variant the relative position of the RT-PCR primers (red arrows), quantitative qRT-PCR (black arrows) and internal probes (blacks lines) used are represented. **B.** Expression of different aromatase transcript variants (T1, T2 and T3) during mouse testis development. Products were generated by RT-PCR with the primer combinations E2-E9/10 (see Table 1) and visualized on 2% agarose gel. NC: 3 negative control (PCR master mix without template). M: DNA ladder; dpc: days post-coitum; dpp: days post-partum. **C.** Absolute quantitative expression of aromatase transcripts during mouse testis development. Total mRNA was isolated from fetal testes at the indicated stages of development, reverse transcribed and aromatase expression was quantified by real-time PCR using the TaqMan method. This method allows the identification of each isoform by using a specific internal probe (see Figure 1A for position and Table 1 for sequences). In order to calculate precisely the copy number of each transcript, each transcript was isolated on gel, re-amplified and quantified. For each experiment a standard curve was constructed using the isolated transcript as template with their corresponding qRT-PCR primers. Data shown are the mean ± SEM (n = 4-6). ^*^ *P* < 0.05 (Student’s *t*-test; compared to the mRNA copy number at 13.5 dpc). **D.** Aromatase protein expression during mouse testis development. On western blots, three protein isoforms were recognized by the anti-aromatase antibody (MCA2077T, Serotec, France). Lane A, ovary from a pregnant mouse: only one band that corresponds to the aromatase full-length protein. Lane B, 2 dpp mouse testis; lane C and D, extracts from 15.5 and 13.5 dpc mouse testes, respectively.

In this paper, we focussed on the ontogenesis of cytochrome P450 aromatase (accession # NP_031836 for protein and NM_007810.3 for mRNA), and named “aromatase” thereafter. To characterize aromatase expression during mouse testis development, its mRNA level was analyzed by RT-PCR from 12.5 dpc to birth. Three transcript variants, called T1, T2 and T3, were detected (Figure 1A-B). T1 and T2 were observed as early as 12.5 dpc. Sequencing confirmed that they were bona fide aromatase variants and showed that they corresponded to full-length aromatase (T1) and to the splicing variants without exon 3 (T2) and without exons 3 and 4 (T3) (see Additional file 1).

In order to quantify these variants during embryogenesis, we performed quantitative real-time RT-PCR (qRT-PCR) using the TaqMan method which allows the detection of each isoform using three different internal probes each one specific for one isoform (Figure 1A and Table 1 for sequences and conditions). We showed that T1 and T2 expression in the testis increased by around 20-fold between 13.5 and 17.5 dpc and thereafter started to progressively decrease (Figure 1C).

**Table 1:** **Sequences of aromatase primers used in RT-PCR and qRT-PCR**

		Sequence 5′-3′	Tm
RT-PCR all transcripts	forward	AACCCCATGCAGTATAATGTC	55°C
	reverse	CATTCTTCTCAAAGTTTTCA	
T1 qRT-PCR	forward	GCCTCCTTCTCCTGATTTGGA	60°C
	reverse	CTGCCATGGGAAATGAGGG	
	internal probe	TACCAGGTCCTGGCTACT	
T2 qRT-PCR	forward	GCCTCCTTCTCCTGAATTTGGA	60°C
	reverse	CCGAATCGGGAGATGTAGTGA	
	internal probe	TCAATACCAGGTCCTCAAGC	
T3 qRT-PCR	forward	CCATGCCACTCCTGCTGAT	60°C
	reverse	CCACCATTCGAACAAGACCAG	
	internal probe	TCTTCAATACCAGCTCTGACGGGCC	

To determine if aromatase is translated in mouse testis, western blot analysis was performed using a specific anti-aromatase antibody (MCA2077T, Serotec, France) (Figure 1D). Two proteins around 54 kDa and one around 27 kDa were detected. The protein of 54 kDa was also present in the ovary extract and it approximately corresponds to the aromatase expected size. We thus suppose that the two heaviest proteins derived from the full-length form of aromatase (T1) with the highest form corresponding to a testis-specific post-translational modification that remains to be identified. In order to understand the origin of the 27 kDa protein, we analysed sequences of the T2 and T3 variants. It revealed that the splicing of exon 3 in T2 would change the ORF and create a precocious codon stop leading to a probably not detected protein of 6 kDa. Splicing of exons 3 and 4 in T3 would not change the ORF allowing in theory the synthesis of a truncated protein of 46 kDa. No protein at this expected size was detected in the western blot (Figure 1D). However, the use of an alternative start codon located later in T2 and T3 sequences may lead to a protein of 27 kDa containing the C-terminal part of aromatase.

These findings are different from those of the only previously published paper on this topic showing that, in the mouse, aromatase expression starts at 17.5 dpc and reaches the highest level at day 1 post-partum [3]. In our study, we detected aromatase expression as early as 12.5 dpc. This discrepancy probably results from the improvement of the methods of detection made since 1994. This is an important point because it shows that estrogens can be produced by mouse fetal testes very early and throughout development.

Our findings indicate that different aromatase transcripts are generated in fetal mouse testes. Previous studies in different mammalian species (including the mouse) reported that tissue-specific aromatase expression is driven by specific promoters [4–6]. Each tissue-specific promoter is associated with a specific untranslated first exon. In mice testis Golovine et al. have shown that aromatase transcripts may emerge from a specific promoter called Ptes [4]. Our study showed that aromatase expression is also regulated at a second transcriptional level generating two additional truncated variants T2 and T3 by mRNA splicing. Our results suggest that there are several forms of aromatase protein however the nature and the physiological function of these isoforms remain to be investigated.

### Aromatase cell localization in mouse fetal testes

**Figure 2:**
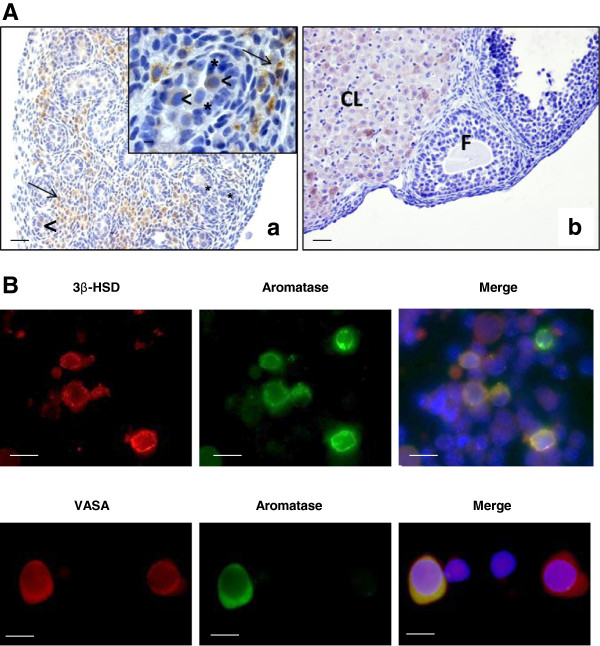
**Aromatase cell localization in mouse fetal testis. A.** Aromatase immunodetection in 17.5 dpc testes. Immunostaining was performed using an anti-aromatase antibody (MCA2077T, Serotec, France), followed by a biotin-conjugated secondary antibody and streptavidin-peroxidase visualization with DAB (Vector Laboratories). **(a)** A strong specific immunoreactivity was observed in Leydig cells within the interstitial tissue (arrows) and to a lesser extent in gonocytes (arrowheads). Inset is a higher magnification to show that aromatase is localized only in the cytoplasm of Leydig cells (arrows) and gonocytes (arrowheads) and is absent or not detectable in the Sertoli cells (asterisks). **(b)** Control section (ovary from adult pregnant rat): positive staining is observed in the corpora lutea (CL) but not in the pre-antral follicle (F), as previously described [13]. Scale bars = 5 μm. **B.** Double immunofluorescence staining for aromatase, 3βHSD (Leydig cell marker) and VASA (germ cell marker) in enriched gonocyte cultures. Immunostaining was performed using anti-aromatase (MCA2077T, Serotec, France) (green), anti-3βHSD (generous gift by J.I Mason) (red) and anti-VASA antibodies (ab13840, Abcam, France) (red). Aromatase expression was detected both in 3βHSD-positive cells and in VASA-positive cells. Nuclei were visualized with 4′-6 diaminido-2-phenylindole (DAPI) (blue). Scale bars = 10 μm.

Immunohistochemical analysis of aromatase localization in 17.5 dpc mouse testes using a specific anti-aromatase antibody (MCA2077T, Serotec, France) showed a strong staining in Leydig cells. Importantly, there was no detectable staining in Sertoli cells (Figure 2A). Conversely, previous studies in fetal and neonatal testes showed that aromatase was expressed in both Leydig cells and Sertoli cells in the rat [7, 8]. This and other previous reports indicate that aromatase cell localization in fetal testis is quite variable in mammalian species. Indeed, aromatase is expressed in Leydig cells and not in Sertoli cells in the fetal testis of the Plains Vizcacha rodent [9], is totally absent in the deer [10], and is detected in both Sertoli cells and Leydig cells in fetal baboon and human testes [11, 12].

In addition, our immunohistochemical analysis showed that aromatase was also expressed in gonocytes, but the intensity of the signal was not uniform: in some cells the signal was very strong, whereas in others it was faint or undetectable (Figure 2A, arrowheads). Similar results were previously described for Retinoic Acid Receptor alpha [14]. As aromatase localization in germ cells was quite unexpected, aromatase immunostaining was also performed in enriched gonocyte cultures that were prepared from 17.5 dpc mouse testes as previously described [15]. Similarly, aromatase was detected in some germ cell VASA-positive cells, a germ cell-specific marker (Figure 2B). This result identifies a sub-population of gonocytes with endocrine function. Aromatase expression was previously reported in adult rat and human germ cells [16, 17] and in pig gonocytes during development [18]. Aromatase expression was also detected in gonocytes of human fetal testes [12].

In conclusion, aromatase cell localization in fetal testis appears to differ from one species to another and as consequence also the intracellular estrogen concentration. These differences should be taken into account to explain the variations in the susceptibility of fetal testis to estrogenic and anti-androgenic endocrine disruptors in different mammalian species that has been recently lightened [2].

## Electronic supplementary material

Additional file 1: **Sequencing results of T1, T2 and T3.** The three different transcripts were isolated on gel and sequenced with the following primers: forward 5′-AACCCCATGCAGTATAATGTC-3′ (located in exon II); reverse 5′-CACAATAGCACTTTCGTCCA-3′ (located in exon V). Each different exon is highlighted in a different color (red exon II, black exon III, and blue exon IV and green exon V. In addition, sequencing from exons VI to X were performed using other primers and showed no difference in T1, T2 and T3 (data not shown). (DOC 24 KB)
