# Supplementary material for: Expression and localization of aromatase during fetal mouse testis development
Source: Basic Clin Androl. 2013 Dec 1;23:12. doi: 10.1186/2051-4190-23-12 (PMC4349472; doi:10.1186/2051-4190-23-12)
Supplement: Supplementary file 1 — Additional file 1: Sequencing results of T1, T2 and T3. The three different transcripts were isolated on gel and sequenced with the following primers: forward 5′-AACCCCATGCAGTATAATGTC-3′ (located in exon II); reverse 5′-CACAATAGCACTTTCGTCCA-3′ (located in exon V). Each different exon is highlighted in a different color (red exon II, black exon III, and blue exon IV and green exon V. In addition, sequencing from exons VI to X were performed using other primers and showed no difference in T1, T2 and T3 (data not shown). (DOC 24 KB) [file 12610_2013_12_MOESM1_ESM.doc]

Aromatase T1 from sequencing (598 pb)

GTCACCATCATGGTCCCGGAAACTGTGACTGTCAGTGCCATGCCACTCCTGCTGATCATGGGCCTCCTTCTCCTGATTTGGAATTGTGAGAGCTCGTCTTCAATACCAGGTCCTGGCTACTGTCTGGGAATTGGGCCCCTCATTTCCCATGGCAGATTCTTGTGGATGGGGATTGGAAGTGCCTGCAACTACTACAATAAGATGTATGGAGAGTTCATGAGAGTCTGGATCAGTGGAGAGGAGACACTCATTATCAGCAAGTCCTCAAGCATGTTCCATGTCATGAAGCACAGTCACTCATCTCCCGATTCGGCAGCAAGCGTGGGCTGCAGTGCATCGGCATGCATGAGAACGGCATCATATTTAACAACAACCCGAGCCTTTGGAGAACAATTCGCCCTTTCTTTATGAAAGCTCTGACGGGCCCTGGTCTTGTTCGAATGGTGGAAGTTTGTGTGGAGTCCATCAAGCAGCATTTGGACCGGCTGGGCGAAGTCACCGACACCTCGGGCTACGTGGATGTGTTGACCCTCATGAGACACATCATGCTGGACACCTCTAACATGCTCTTCCTGGGGATCCCCCTGGACGAAAG

Aromatase T2 from sequencing (392 pb)

GTGACTGTCAGTGCCATGCCACTCCTGCTGATCATGGGCCTCCTTCTCCTGATTTGGAATTGTGAGAGCTCGTCTTCAATACCAGGTCCTCAAGCATGTTCCATGTCATGAAGCACAGTCACTACATCTCCCGATTCGGCAGCAAGCGTGGGCTGCAGTGCATCGGCATGCATGAGAACGGCATCATATTTAACAACAACCCGAGCCTTTGGAGAACAATTCGCCCTTTCTTTATGAAAGCTCTGACGGGCCCTGGTCTTGTTCGAATGGTGGAAGTTTGTGTGGAGTCCATCAAGCAGCATTTGGACCGGCTGGGCGAAGTCACCGACACCTCGGGCTACGTGGATGTGTTGACCCTCATGAGACACATCATGCTGGACACCTCTAACATG

Aromatase T3 from sequencing (309 pb)

AACCCCATGCAGTATAATGTCACCATCATGGTCCCGGAAACTGTGACTGTCAGTGCCATGCCACTCCTGCTGATCATGGGCCTCCTTCTCCTGATTTGGAATTGTGAGAGCTCGTCTTCAATACCAGCTCTGACGGGCCCTGGTCTTGTTCGAATGGTGGAAGTTTGTGTGGAGTCCATCAAGCAGCATTTGGACCGGCTGGGCGAAGTCACCGACACCTCGGGCTACGTGGATGTGTTGACCCTCATGAGACACATCATGCTGGACACCTCTAACATGCTCTTCCTGGGGATCCCCCTGGACGAA
